# Anisotropic CdSe Tetrapods in Vortex Flow for Removing Non-Specific Binding and Increasing Protein Capture

**DOI:** 10.3390/s22155929

**Published:** 2022-08-08

**Authors:** Hanzhe Liu, Dong June Ahn

**Affiliations:** 1 Department of Chemical and Biological Engineering, Korea University, Seoul 02841, Korea; 2 KU-KIST Graduate School of Converging Science and Technology, Korea University, Seoul 02841, Korea

**Keywords:** anisotropic nanoparticles, CdSe tetrapods, protein capture, non-specific binding removal, cyclic mode vortex flow

## Abstract

Non-specific binding (NSB) is one of the important issues in biosensing performance. Herein, we designed a strategy for removing non-specific binding including anti-mouse IgG antibody and bovine serum albumin (BSA) by utilizing anisotropic cadmium selenide tetrapods (CdSe TPs) in a vortex flow. The shear force on the tetrapod nanoparticles was increased by controlling the rotation rate of the vortex flow from 0 rpm to 1000 rpm. As a result, photoluminescence (PL) signals of fluorescein (FITC)-conjugated protein, anti-mouse IgG antibody-FITC and bovine serum albumin (BSA)-FITC, were reduced by 35% and 45%, respectively, indicating that NSB can be removed under vortex flow. In particular, simultaneous NSB removal and protein capture can be achieved even with mixture solutions of target antibodies and anti-mouse IgG antibodies by applying cyclic mode vortex flow on anisotropic CdSe TPs. These results demonstrate successfully that NSB can be diminished by rotating CdSe TPs to generate shear force under vortex flow. This study opens up new research protocols for utilization of anisotropic nanoparticles under vortex flow, which increases the feasibility of protein capture and non-specific proteins removal for biosensors.

## 1. Introduction

The application of protein capture has attracted considerable interest in disease diagnosis, and studies have focused on improving sensitivity and selectivity of the biosensors [1,2,3]. Various technologies, such as surface plasmon resonance and enzyme-linked immunosorbent assays [4,5,6,7,8,9], have been applied to the field of protein capture. The technologies generated specific signals for target analyte via interaction between probe and target proteins. To further increase sensitivities of these biosensors, non-specific binding of proteins should be avoided. Non-specific proteins induced by the physical, chemical, or electrostatic adsorption [10,11] can result in a false signal, a decrease in the signal-to-noise ratio, and a reduction in the sensitivity and selectivity of protein capture.

Separation techniques, including desalting and filtration, can be employed to reduce non-specific binding (NSB) of proteins [12,13,14]. Another way to overcome non-specific adsorption is by utilizing buffer solutions, such as Tween-20 and sodium dodecyl sulfate. This approach creates a hydrophilic and noncharged interface layer to reduce protein adsorption [15,16,17,18,19,20,21]. In addition, microfluidic devices have also been applied to remove non-specific protein adsorption by manipulating surface shear forces and fluid mixing. The shear force occurs due to the friction between the nanoparticle and the aqueous fluid flow. It enables the preferential selection of strongly bound specific proteins and thus enhances target signals [22,23,24,25]. Most microfluidic devices for protein capture have focused on the nanoparticles at the constant flow rate. However, little attention has been paid to utilization of the morphological design of nanoparticles under flow field for improving protein capture.

To date, nanomaterials with various morphologies, such as quantum dots, nanorods, nanowires, and nanoplates have been applied to increase protein capture [26,27,28,29,30,31,32,33,34]. Herein, we report a new approach for capturing proteins by rotating tetrapod nanoparticles in a vortex flow. Cadmium selenide tetrapods (CdSe TPs) were prepared as probe nanoparticles for protein capture. Rotating CdSe TPs by the vortex flow can generate a shear force on the surface of nanoparticles and enhance the target protein captures [23] and removal of non-specific proteins [35]. In this experiment, we applied the vortex flow on CdSe TPs to simultaneously capture and remove proteins. The rotation rate and mode, including cyclic mode and continuous mode, were controlled to increase induced shear force. Thus, simultaneous protein removal and protein capture can be achieved in mixed solutions, which is greatly improved after six cycles of static and vortex flow.

## 2. Materials and Methods

Materials. CdSe TPs were prepared as previously described [36,37]. Bovine serum albumin (BSA) (≥98%), goat anti-human IgG antibody, fluorescein isothiocyanate (FITC)-conjugated rabbit anti-goat IgG, rabbit anti-goat IgG, FITC-conjugated rabbit anti-mouse IgG, rabbit anti-mouse IgG, BSA-FITC, 3-mercaptopropionic acid (MPA, ≥99%, HPLC), N-(3-dimethylaminopropyl)-N-ethylcarbodiimide hydrochloride (EDC, ≥99%), N-hydroxysuccinimide (NHS, ≥99%), 2-(N-Morpholino)ethanesulfonic acid (MES, ≥99%), sodium phosphate monobasic (NaH_2_PO_4_, ≥99%), sodium phosphate dibasic (Na_2_HPO_4_, ≥99%), sodium chloride (NaCl, ≥99%), chloroform (≥99%), and ethanol (≥95%), were purchased from Sigma-Aldrich.

Functionalization of nanoparticles with a primary antibody. CdSe TPs mixed with MPA were incubated overnight in chloroform. The solution was rinsed and centrifuged at 6000 rpm. The obtained CdSe TPs were stored in deionized water. A primary antibody (goat anti-human IgG antibody, 0.1 nM) was added to the CdSe TPs solution, which was then added to EDC (0.1 M)/NHS (0.2 M) in an MES buffer (0.1 M, pH = 5.5). The solution was shaken for 6 h. The primary antibody-CdSe TPs (Ab-CdSe TPs) were rinsed (three times) and stored in phosphate-buffered saline (PBS) (pH 7.4: 0.562 g Na_2_HPO_4_, 0.125 g NaH_2_PO_4_, 4.383 g NaCl, 500 mL deionized water) at 4 °C.

Targets binding of antibody functionalized nanoparticles. Ab-CdSe TPs were extracted and mixed with anti-goat IgG antibody, anti-mouse IgG antibody, and BSA labelled with FITC, respectively, to form three types of complexes. The complexes were formed after shaking the mixture overnight (12 h) at 25 °C. The concentration of each protein was 0.1 nM. Then, CdSe TP complexes were centrifuged (6000 rpm, 10 min) and rinsed (three times).

Removal of the anti-mouse IgG antibody and BSA in the vortex flow. The CdSe TP complexes were rotated at 400, 600, 800, and 1000 rpm for 30 min. The samples were centrifuged, rinsed, and then added to PBS.

Protein capture in the vortex flow. The Ab-CdSe TPs were extracted and mixed with anti-goat IgG antibody in the PBS blocking buffer (pH 7.4: 0.562 g Na_2_HPO_4_, 0.125 g NaH_2_PO_4_, 4.383 g NaCl, 500 mL deionized water, 0.1% BSA wt/vol). The mixture was rotated at 1000 rpm for 30 min. The CdSe TP complexes were then centrifuged (6000 rpm, 10 min) and rinsed (three times).

Protein capture in the mixture solutions. Two samples of mixture solution with anti-goat IgG antibody-FITC and anti-mouse IgG antibody, and with anti-goat IgG antibody and anti-mouse IgG antibody-FITC were prepared to calculate the occupancy of antibodies on complexes. Total protein concentration was 0.1 nM. We used mixture solutions with different concentration ratios. The concentration ratios of anti-goat IgG antibody: anti-mouse IgG antibody were 50%:50%, 40%:60%, 25%:75%, and 10%:90%. The mixture was rotated at 1000 rpm for 30 min. The CdSe TP complexes were then centrifuged (6000 rpm, 10 min) and rinsed (three times).

Experiments of CdSe TPs in the mixture solutions under cycle mode vortex flow. For one cycle of the cyclic mode vortex flow, CdSe TP complexes were rotated for 30 min and subsequently remained static for 30 min. After conducting cyclic mode vortex flow for 1~6 cycles, the complexes were centrifuged and washed.

Quantitative analysis of protein capture and NSB removal. We calculated the content of protein captured or removed by comparing PL intensity of FITC labelled on antibody, as follows:

$$\Delta \mathrm{PL}=\frac{\left|{\mathrm{PL}}_{2}-{\mathrm{PL}}_{1}\right|}{{\mathrm{PL}}_{1}}\times 100\%$$

PL_2_ represents the PL intensity of anti-goat IgG antibody-FITC or anti-mouse IgG antibody-FITC after the rotation and PL_1_ represents the PL intensity of anti-goat IgG antibody-FITC or anti-mouse IgG antibody-FITC before the rotation.

Characterization of the CdSe TP samples. The fluorescence spectra were recorded using a HITACHI F-7000 (Tokyo, Japan) with an excitation at 488 nm, and the emission was measured from 500 nm to 700 nm in 1-nm increments. Fluorescence microscopy images were obtained with an Olympus microscope (Tokyo, Japan). Powdered samples of CdSe TP were cast on cover glass. The images were recorded with an exposure time of 1/5 s. The morphology images were acquired using transmission electron microscopy (Tecnai G2, Fei). Powdered samples of CdSe TP were cast on an ultrathin carbon-coated Cu grid. The images were captured with an acceleration voltage of 200 kV. An MB-102 mixing block was used (Seoul, Korea).

## 3. Results and Discussion

**Figure 1:**
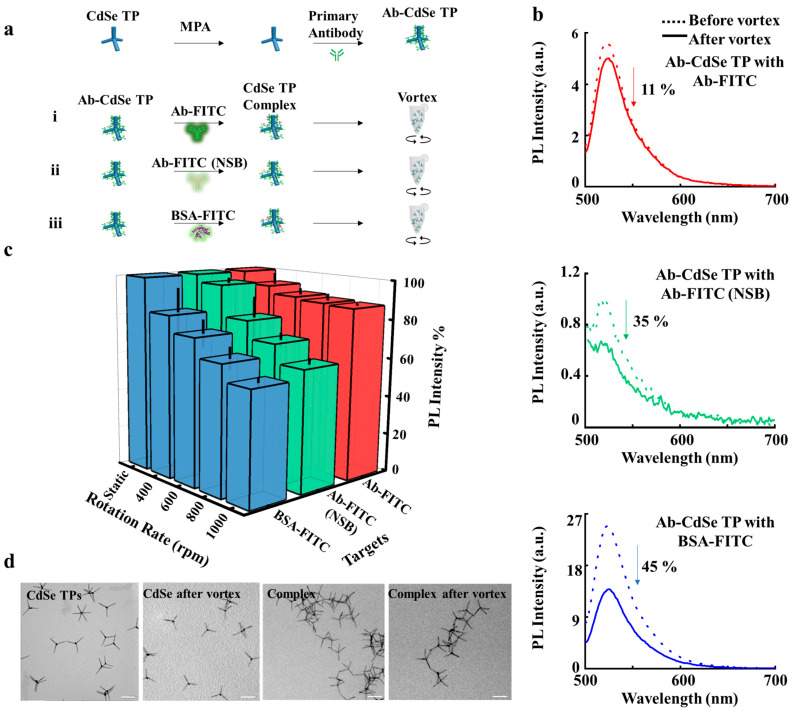
CdSe TP complexes with various proteins under vortex flow. (**a**), Schematic of the CdSe TPs functionalized with the primary antibodies, binding with anti-goat IgG antibody, anti-mouse IgG antibody, and BSA, respectively. The CdSe TP complexes before and after rotation was used for the photoluminescence signal analysis. (**b**), PL intensities of the CdSe TP complexes including the anti-goat IgG antibody (**top**), anti-mouse IgG antibody (**middle**), and BSA (**bottom**) before and after rotation. (**c**), PL intensities as a function of the rotation rate, including 0, 400, 600, 800 and 1000 rpm, respectively. The average values are based on data collected from more than 3 samples prepared independently; the error bar represents the standard deviation of the PL intensity data. (**d**), TEM images of the CdSe TPs before and after the vortex flow. Scale bar, 100 nm.

A schematic illustration of protein removal by vortex flow is shown in Figure 1. The anti-human IgG (primary antibody) was immobilized on CdSe TPs to capture anti-goat IgG antibody, anti-mouse IgG antibody (NSB), and bovine serum albumin (BSA) labelled with FITC fluorescent dyes. The PL spectra and fluorescence images of different complexes were observed to analyze the protein removal, as shown in Figure S1. PL spectra show a broad peak at 520 nm, the main PL peak of FITC [38], when complexes are excited at 488 nm, which corresponds to the main absorption band of FITC. The anti-goat IgG antibodies are captured on the Ab-CdSe TPs by approximately 6-fold more than the anti-mouse IgG antibodies (Figure S1). The high PL intensity at 520 nm of BSA-FITC (Figure S1) indicates that more BSAs are captured by the Ab-CdSe TPs by approximately 2.4-fold compared to the anti-goat IgG antibody, as the BSA can non-specifically bind to the surface of Ab-CdSe TPs. Isothermal titration calorimetry analyses of anti-goat IgG antibody and anti-mouse IgG antibody upon protein capture, shown in Figure S2, indicate that antibody specifically binds to primary antibody, whereas anti-mouse IgG antibodies non-specifically adsorb on it [39]. Then we measured the PL intensities of the proteins on complexes before and after applying vortex flow, as shown in Figure 1b. The PL intensity of anti-goat IgG antibody is slightly reduced by 11% (Figure 1b, top) after vortex flow. In contrast, the PL intensities of anti-mouse IgG antibody and BSA that form non-specific binding are reduced by 35% (Figure 1b, middle) and 45% (Figure 1b, bottom) after the vortex flow. Subsequently, the shear force was increased to remove protein [23] by controlling the rotation rate, which was controlled to evaluate the effects on protein removal, as shown in Figure 1c. The PL intensity of anti-goat IgG antibody is slightly reduced with respect to rotation rates. In contrast, the PL intensities of both anti-mouse IgG antibody and BSA are strikingly reduced after increasing rotation rates. Morphological analyses for CdSe TP complexes before and after rotation were conducted by resolution transmission electron microscopy (TEM) to observe whether CdSe TPs were damaged to cause the loss of fluorescence. In Figure 1d, the CdSe TP complexes maintain their structures having arms with a uniform diameter of 3 nm and length of 60 nm before and after applying vortex flow, indicating the loss of fluorescence is mainly induced by the removal of antibody labeled with FITC. Thus, as the PL intensities are strikingly reduced for anti-mouse IgG antibody and BSA compared to anti-goat IgG antibody, we can distinguish specific binding from non-specific binding and remove non-specific proteins by applying vortex flow on anisotropic CdSe TPs. 

**Figure 2:**
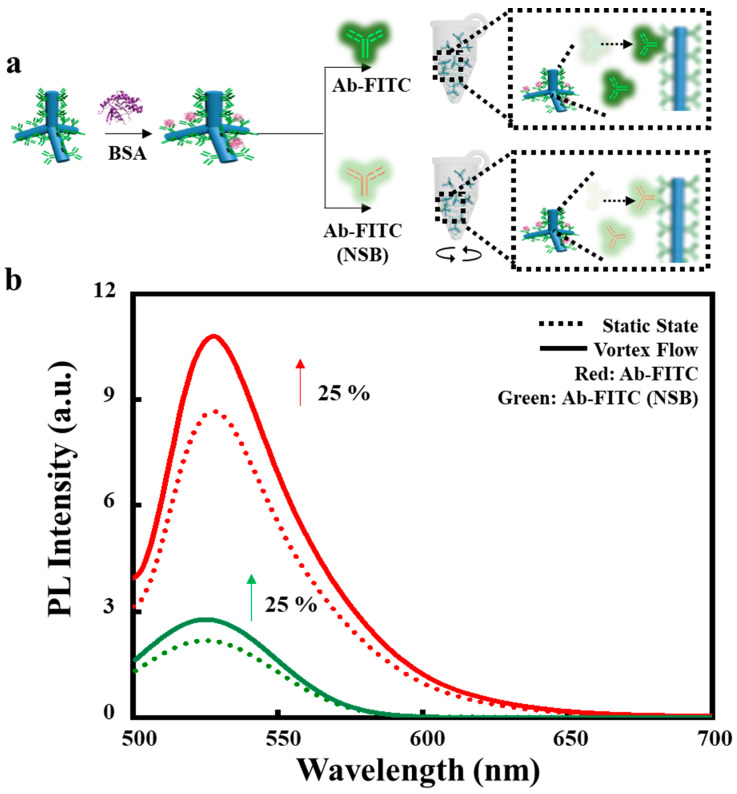
Protein capture before and after rotation. (**a**), Schematic of the CdSe TP functionalized with the primary antibody and BSA as a blocking agent. The PL intensities were compared to evaluate complexes before and after vortex flow. (**b**), PL spectra of the complexes with anti-goat IgG antibody and anti-mouse IgG antibody before and after vortex flow. Rotation time, 30 min. Rotation rate, 1000 rpm.

In the next step, we measured the content of captured proteins on CdSe TPs under vortex flow, as shown in Figure 2. CdSe TPs were functionalized with primary antibody and blocked by BSA preventing non-specific binding. PL spectra of CdSe TPs upon adding anti-goat IgG antibody and anti-mouse IgG antibody are shown in Figure 2b. PL intensity from CdSe TPs upon binding with antibody indicates that more anti-goat IgG antibodies are captured on CdSe TPs via specific binding compared to adsorbing anti-mouse IgG antibodies. Rotating complexes to generate flow motion, and increased shear force can promote convection, which ensures the enhanced protein capture at 1000 rpm. PL intensities of both complexes with anti-goat IgG antibody and anti-mouse IgG antibody increase by approximately 1.25-fold. From these results, we deduce that protein capture can be increased by rotating [26] CdSe TPs in a vortex flow.

**Figure 3:**
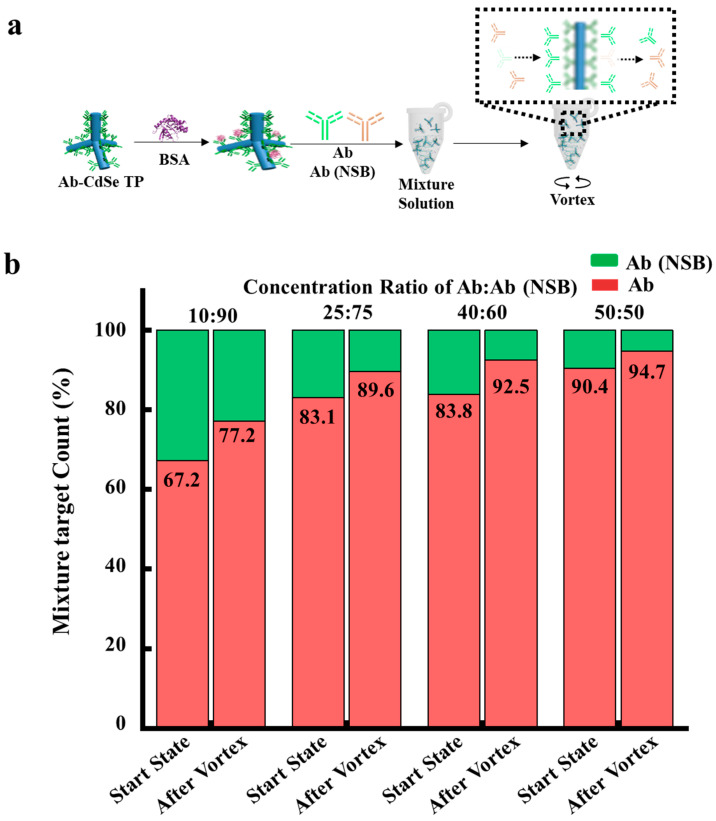
Analysis for the signal changes of the complexes in the mixture solutions before and after rotation. (**a**), Schematic of the CdSe TP complexes with both anti-goat IgG antibody and anti-mouse IgG antibody before and after rotation at concentration ratios of 50%:50%, 40%:60%, 25%:75%, and 10%:90%. (**b**), Occupancy of anti-goat IgG antibody-FITC (red) and anti-mouse IgG antibody-FITC (green) on CdSe TP complexes before and after rotation at various concentration ratios. Occupancy of antibodies were calculated from PL signal from FITC. Reaction time, 30 min. Rotation rate, 1000 rpm.

Then, we explored the capability of capturing target proteins and removing non-specific proteins in mixture solutions, as shown in Figure 3. We selected four mixture solutions with the concentration ratios of anti-goat IgG antibody and anti-mouse IgG antibody at 10%:90%, 25%:75%, 40%:60% and 50%:50%, respectively. Two samples of mixture with (i) FITC labelled anti-goat IgG antibody and anti-mouse IgG antibody and with (ii) FITC labelled anti-mouse IgG antibody and anti-goat IgG antibody were prepared to calculate the amount of captured anti-goat IgG antibody and anti-mouse IgG antibody on Ab-CdSe TPs. Figure 3b shows occupancy of anti-goat IgG antibodies and anti-mouse IgG antibodies calculated from fluorescence intensities on the complex before and after the vortex flow to generate shear force. The occupancy of anti-goat IgG antibodies captured at complexes increases after the vortex flow from 67.2% to 77.2% in a concentration ratio of 10%: 90%. The occupancy of anti-goat IgG antibodies after the vortex flow also increases at high concentration ratio of 50%: 50% from 90.4% to 94.7%. PL intensity of anti-mouse IgG antibody-FITC on CdSe surface is decreased, indicating that the non-specific proteins on a nanoparticle surface is removed by applying vortex flow to generate shear force in mixture solutions. These results demonstrate that simultaneous anti-goat IgG antibody capture and anti-mouse IgG antibody removal can be achieved by applying vortex flow to generate shear force in the mixture solution, thus increasing the sensitivity of CdSe TP complexes.

**Figure 4:**
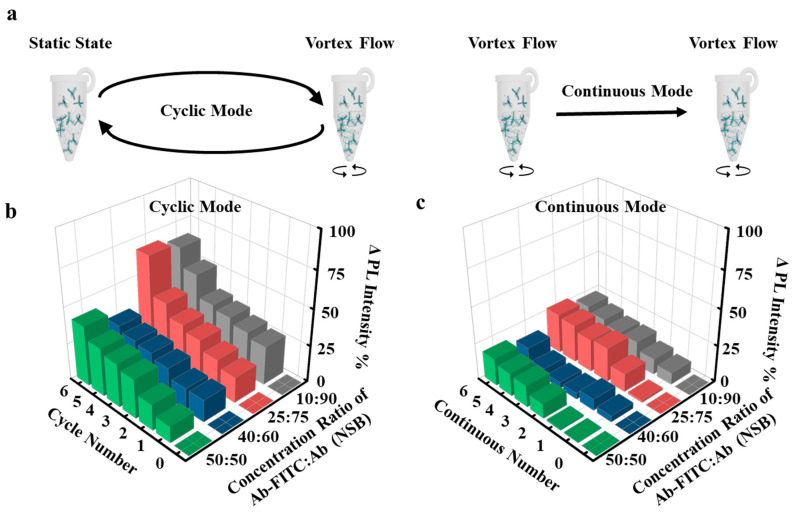
Protein removal and protein capture in the mixture solutions under continuous mode and cyclic mode vortex flow. (**a**), Schematic of the CdSe TP complexes under cyclic mode vortex flow, rotating for 30 min and subsequently remaining static for 30 min; the cycles were repeated for six times. (**b**), PL intensity as a function of the number of cycles for cyclic-mode vortex flow. (**c**), PL intensity as a function of the time for continuous-mode vortex flow. The CdSe TP complexes under continuous mode vortex flow were rotated for 6 h. Rotation rate: 1000 rpm.

To further increase the sensitivity of complexes in the mixture solution, we designed experiments with continuous mode vortex flow and cyclic mode vortex flow. Continuous mode vortex flow rotated complexes for 1 h, whereas cyclic mode vortex flow rotated complexes for 30 min and remained static for 30 min in one cycle (Figure 4b). PL intensities of samples with different concentration ratios of 10%: 90%, 25%: 75%, 40%: 60% and 50%: 50% increase by 22.8%, 27.3%, 14.6% and 18.7% after 6 h of continuous mode, respectively (Figure 4c), indicating that more anti-goat IgG antibodies are captured on complexes under continuous mode vortex flow. In particular, PL intensities of samples with four different concentration ratios were increased even more by 64.7%, 68.3%, 30.4% and 41.1%, respectively, after six cycles of cycle mode vortex flow than after 6 h of continuous mode vortex flow. Thus, we conclude that the cyclic mode vortex flow ensures the non-specific protein removal and target protein capture more efficiently.

## 4. Conclusions

In summary, simultaneous protein capture and non-specific protein removal is successfully achieved by rotating anisotropic CdSe TPs in the vortex flow. By increasing rotation rates, capabilities of tetrapods for capturing target proteins and removing non-specific proteins are enhanced. PL intensities of complexes with anti-mouse IgG antibody and BSA are reduced by 35% and 45%, respectively, after increasing the rotation rate up to 1000 rpm. Furthermore, abilities of complexes to capture anti-goat IgG antibodies and anti-mouse IgG antibodies are also increased by 25% upon rotating at 1000 rpm. Finally, simultaneous non-specific protein removal and protein capture is confirmed even at mixture solutions of anti-goat IgG antibodies and anti-mouse IgG antibodies by applying cyclic mode vortex flow on anisotropic CdSe TPs based sensing platform. This study opens up new research protocols for utilization of tetrapod nanoparticles under vortex flow, which enables both the increase of protein capture and the removal of non-specific proteins for biosensing.

## Data Availability

The data presented in this study are available on request from the corresponding author. The data are not publicly available due to patient privacy.

## Supplementary Materials

The following supporting information can be downloaded at: https://www.mdpi.com/article/10.3390/s22155929/s1, Figure S1: CdSe TP complexes with various proteins.; Figure S2: Isothermal titration calorimetry (ITC) analyses of probe-target complex.
