# Supplementary material for: Anisotropic CdSe Tetrapods in Vortex Flow for Removing Non-Specific Binding and Increasing Protein Capture
Source: Sensors (Basel). 2022 Aug 8;22(15):5929. doi: 10.3390/s22155929 (PMC9371395; doi:10.3390/s22155929)
Supplement: Supplementary file 1 [file sensors-22-05929-s001.zip › sensors-1736766-supplementary.pdf]

Supplementary Information for

Anisotropic CdSe Tetrapods in Vortex Flow for Removing Non-Specific Binding and Increasing Protein Capture

Hanzhe Liu[1] and Dong June Ahn[1,2*]

[1]Department of Chemical and Biological Engineering, Korea University, 02841, Seoul, Korea

[2]KU-KIST Graduate School of Converging Science and Technology, Korea University, 02841, Seoul, Korea

* *Correspondence and requests for materials should be addressed to D.J.A. (email: ahn@korea.ac.kr).*

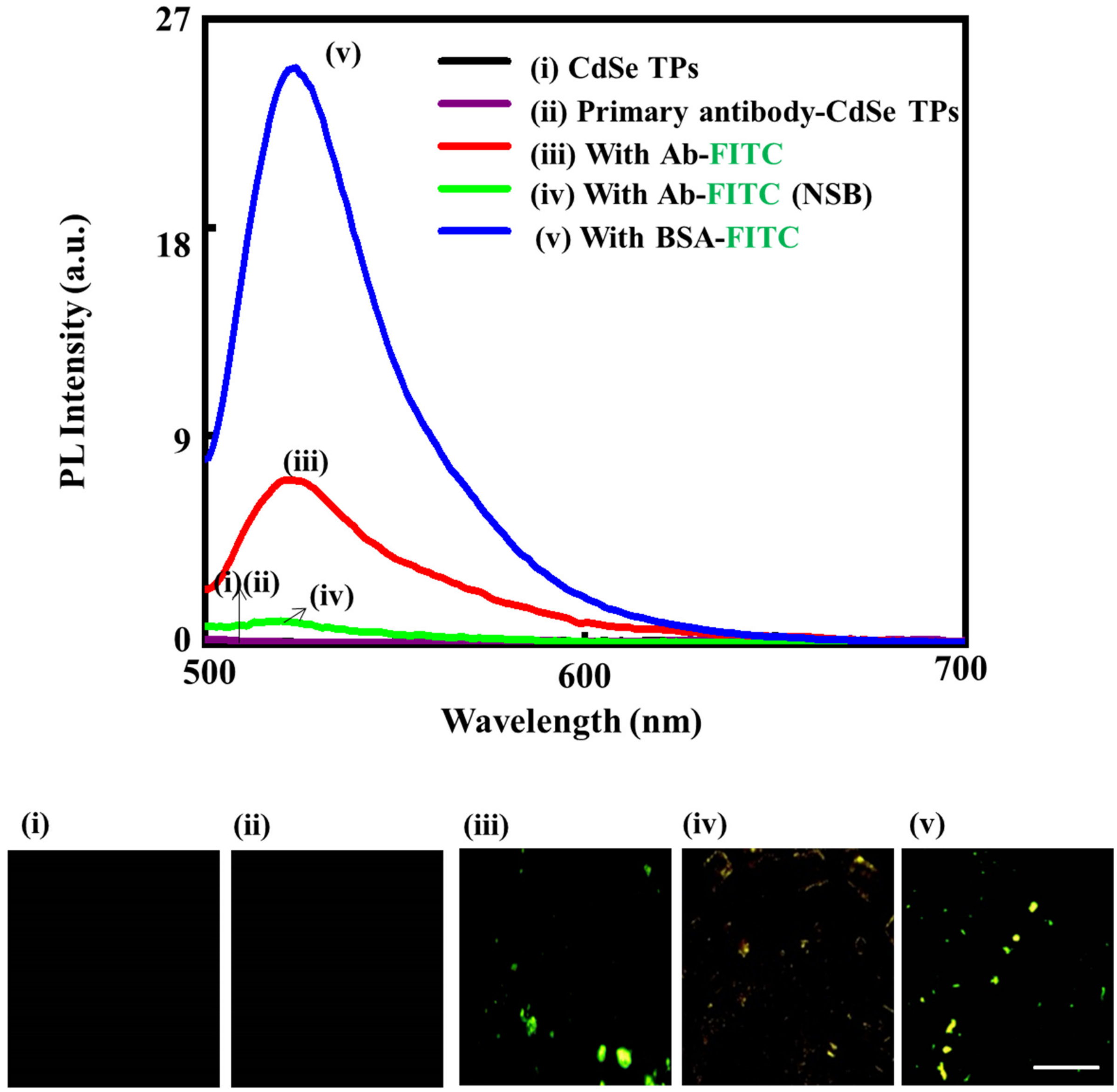

Supplementary Fig. S1 CdSe TP complexes with various proteins. a, PL spectra of CdSe TPs, modified with different antibodies at 488 nm excitation. **b**, The images acquired under a fluorescence microscope present CdSe TPs (i), Ab-CdSe TPs (ii), Ab-CdSe TPs with antibody (iii), Ab-CdSe Tps with Antibody (NSB) (v), and Ab-CdSe TPs with BSA (iv). Scale bar is 5 μm.

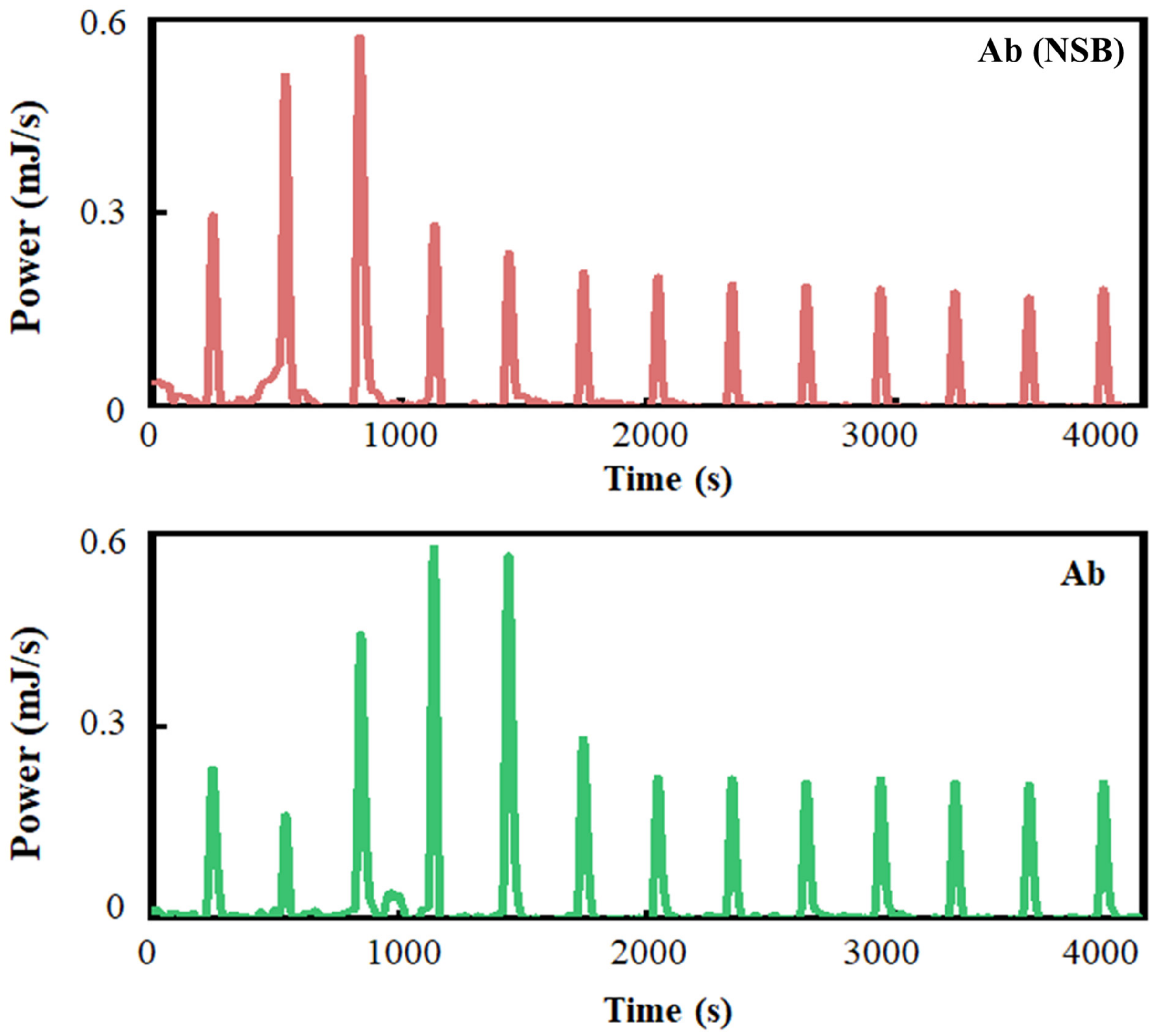

Supplementary Fig. S2 Isothermal titration calorimetry (ITC) analyses of probe-target complex. a, ITC measurement between antibody (NSB) and probe Ab. **b**, ITC measurement between target antibody and probe Ab.
